# Increased Endogenous GDNF in Mice Protects Against Age-Related Decline in Neuronal Cholinergic Markers

**DOI:** 10.3389/fnagi.2021.714186

**Published:** 2021-08-12

**Authors:** Sumonto Mitra, Giorgio Turconi, Taher Darreh-Shori, Kärt Mätlik, Matilde Aquilino, Maria Eriksdotter, Jaan-Olle Andressoo

**Affiliations:** ^1^Division of Clinical Geriatrics, Center for Alzheimer Research, Department of Neurobiology, Care Sciences and Society (NVS), Karolinska Institutet, Huddinge, Sweden; ^2^Department of Pharmacology, Faculty of Medicine and Helsinki Institute of Life Science, University of Helsinki, Helsinki, Finland; ^3^Theme Inflammation and Aging, Karolinska University Hospital, Huddinge, Sweden; ^4^Division of Neurogeriatrics, Center for Alzheimer Research, Department of Neurobiology, Care Sciences and Society (NVS), Karolinska Institutet, Stockholm, Sweden

**Keywords:** glial cell line-derived neurotrophic factor (GDNF), nerve growth factor (NGF), aging, cholinergic markers, cholinergic index, choline acetyltransferase (ChAT), acetylcholinesterase (AChE), brain

## Abstract

Gradual decline in cholinergic transmission and cognitive function occurs during normal aging, whereas pathological loss of cholinergic function is a hallmark of different types of dementia, including Alzheimer’s disease (AD), Lewy body dementia (LBD), and Parkinson’s disease dementia (PDD). Glial cell line-derived neurotrophic factor (GDNF) is known to modulate and enhance the dopamine system. However, how endogenous GDNF influences brain cholinergic transmission has remained elusive. In this study, we explored the effect of a twofold increase in endogenous GDNF (Gdnf hypermorphic mice, *Gdnf*^wt/hyper^) on cholinergic markers and cognitive function upon aging. We found that *Gdnf*^wt/hyper^ mice resisted an overall age-associated decline in the cholinergic index observed in the brain of *Gdnf*^wt/wt^ animals. Biochemical analysis revealed that the level of nerve growth factor (NGF), which is important for survival and function of central cholinergic neurons, was significantly increased in several brain areas of old *Gdnf*^wt/hyper^ mice. Analysis of expression of genes involved in cholinergic transmission in the cortex and striatum confirmed modulation of cholinergic pathways by GDNF upon aging. In line with these findings, *Gdnf*^wt/hyper^ mice did not undergo an age-related decline in cognitive function in the Y-maze test, as observed in the wild type littermates. Our results identify endogenous GDNF as a potential modulator of cholinergic transmission and call for future studies on endogenous GDNF function in neurodegenerative disorders characterized by cognitive impairments, including AD, LBD, and PDD.

## Introduction

Glial cell line-derived neurotrophic factor (GDNF) is a secretory protein which protects dopaminergic neurons *in vitro* and *in vivo* (Lin et al., 1993; Hoffer et al., 1994). In preclinical models, GDNF application potentiates striatal dopaminergic fiber outgrowth and elevates striatal dopamine levels (Hoffer et al., 1994; Tomac et al., 1995; Gash et al., 1996; Kordower et al., 2000; Ibanez and Andressoo, 2017). For this reason, intracranial ectopic delivery of GDNF has been tested in several clinical trials for Parkinson’s disease (PD), with encouraging yet inconclusive results (Barker et al., 2020). In the brain, GDNF is predominantly expressed in striatal parvalbumin-positive (PV^+^) and cholinergic interneurons (Hidalgo-Figueroa et al., 2012), although low levels of GDNF have also been reported in other areas of the brain, including the cortex, hippocampus, olfactory bulb, cerebellum and spinal cord (Trupp et al., 1997; Golden et al., 1998). Striatal PV^+^ interneurons form dendrodendritically connected network of cells which regulate the whole striatal output by medium spiny neurons (MSNs), that make up most of the striatal neuronal population (Koos and Tepper, 1999; Kreitzer, 2009; Lee et al., 2017). The striatal dopaminergic signaling is also regulated by striatal cholinergic interneurons (Cachope and Cheer, 2014; Sulzer et al., 2016), which primarily control reward and addiction related behaviors (Berlanga et al., 2003; Gonzales and Smith, 2015; Stouffer et al., 2015), but can also influence cognition (Ztaou et al., 2018; Galaj et al., 2019), social behavior (Martos et al., 2017), behavioral flexibility (Ragozzino et al., 2002, 2009; McCool et al., 2008) and motor function (Pisani et al., 2007).

The striatal interneurons are known to locally express nerve growth factor (NGF; Bizon et al., 1999), the master cholinergic signaling regulator. Additionally, external cholinergic inputs into the striatum originates from the brain stem (Dautan et al., 2014). NGF plays a significant role in cognition and reduction in NGF levels upon aging is known to be associated with neurological conditions, such as Alzheimer’s disease (AD; Mitra et al., 2019). AD is characterized by severely reduced cognitive ability, where loss in spatial memory is an early feature (Cuello et al., 2019; Mitra et al., 2019; Mufson et al., 2019). Cholinergic dysfunction and cognitive impairment also occur in other conditions associated with dementia, including Lewy body dementia (LBD) and Parkinson’s disease dementia (PDD; Simard et al., 2000; Francis and Perry, 2007; Halliday et al., 2014; Perez-Lloret and Barrantes, 2016).

Ectopic NGF application improves spatial memory in aged rats (Fischer et al., 1987, 1991; Markowska et al., 1994; Chen et al., 1995) and rescues spatial learning and memory deficits in brain-specific *Ngf* knock-out mice (Eu et al., 2021). However, studies on GDNF’s effect on spatial memory are scarce. Lentiviral delivery of recombinant human GDNF into the hippocampus of aged rats was shown to improve cognitive function and choline acetyltransferase (ChAT) activity, the acetylcholine (ACh) biosynthesizing enzyme (Pertusa et al., 2008). Whereas reduced level of endogenous GDNF in Gdnf knockout (KO) heterozygous mice affected spatial learning in the Morris water maze test (Gerlai et al., 2001).

Previously, we generated a mouse model where the replacement of the *Gdnf’s* native 3′untranslated region (3′UTR) with a 3′UTR which is less responsive to negative regulators, such as microRNAs, resulted in about a twofold increase in endogenous GDNF expression (GDNF hypermorphic mice, *Gdnf*^wt/hyper^) (Kumar et al., 2015). We found that *Gdnf*^wt/hyper^ mice have about 15% increase in the number of dopaminergic neurons in the substantia nigra and increased nigrostriatal dopaminergic transmission and motor function lasting until old age. Notably, side effects associated with ectopic GDNF delivery and/or enhanced brain dopaminergic function were not observed, suggesting that increase in endogenous GDNF expression is safe and may therefore have potential clinical application (Kumar et al., 2015; Matlik et al., 2018; Turconi et al., 2020).

However, while the role of GDNF as a modulator of the dopaminergic system is well established, the effect of GDNF on the cholinergic system has remained elusive. In this study, we conducted an explorative biochemical and neurobehavioral analysis on young and old *Gdnf*^wt/hyper^ and gender matched littermate control (*Gdnf*^wt/wt^) mice to investigate whether an about twofold increase in endogenous GDNF modulates cholinergic transmission and cognitive functions upon aging.

## Materials and Methods

### Animals

Mice were maintained in a 129Ola/ICR/C57bl6 mixed genetic background. The mice were maintained in temperature-controlled conditions at 20°C–22°C under a 12-h/12-h light/dark cycle at relative humidity of 50–60%. Cages and bedding material (Aspen chips; Tapvei Oy, Finland) were changed every week, and wooden tube and aspen shavings were provided as enrichment. Mice received food and water *ad libitum*. Mice were tested at 2–3 months of age (young) and 15–16 months of age (old). Wild-type littermates (*Gdnf*^wt/wt^) were used as controls in all experiments. Considering that the oestrus cycle is believed to enhance experimental variation and because of the considerable costs of aging studies, we analyzed only male mice in this study. Altogether, three independent cohorts of young and old mice were used in this study. The number of mice in each cohort was as follows: cohort 1 (10 young *Gdnf*^wt/wt^ + 10 young *Gdnf*^wt/hyper^ and 8 old *Gdnf*^wt/wt^ + 8 old *Gdnf*^wt/hyper^), cohort 2 (6 young *Gdnf*^wt/wt^ + 7 young *Gdnf*^wt/hyper^ and 5 old *Gdnf*^wt/wt^ + 6 old *Gdnf*^wt/hyper^), cohort 3 (7 young *Gdnf*^wt/wt^ + 6 young *Gdnf*^wt/hyper^ and 5 old *Gdnf*^wt/wt^ + 12 old *Gdnf*^wt/hyper^). The experiments performed in each cohort were as follows: cohort 1 (enzyme activity assay and NGF protein level); cohort 2 (gene expression and correlation analysis); cohort 3 (behavioral test). More detailed description of the experiments performed is given below.

### Tissue Isolation

For quantitative PCR, mice were euthanized by cervical dislocation followed by decapitation after deep anesthesia with CO_2_. The brain was quickly removed from the skull, immersed in ice-cold PBS, and placed in an ice-cooled brain block (Stoelting, Wood Dale, IL, United States). Brain regions of interest were collected using a puncher (inner diameter, 2 mm) or a scalpel, snap frozen, and stored at −80°C until processed.

For measurement of cholinergic enzymes activity and NGF protein measurement, mice were anesthetized with sodium pentobarbital (100 mg/kg intraperitoneally [i.p.]) and intracardially perfused with PBS to wash out blood from the brain tissue associated blood vessels. After perfusion, the brain was removed from the skull and brain regions collected as described above.

Cholinergic neurons from the basal forebrain are known to innervate multiple cortical regions (Li et al., 2018). These areas include the parietal cortex, which mainly regulate supramodal attention (Ljubojevic et al., 2018) and the prefrontal cortex, important regulator of cognitive functions (Miller, 2000; Horst and Laubach, 2009). A previous study suggested that prefrontal cortex can also modulate the release of ACh in the parietal cortex (Nelson et al., 2005). In this study, enzyme activity assays and NGF protein measurements were performed from parietal cortex tissues, whereas gene expression and correlation analyses were performed from prefrontal cortex tissues. Additionally, other brain regions like basal forebrain, striatum and hippocampus were also dissected as described in Kumar et al. (2015) from cohort 1 using a mouse brain atlas as a reference. Tissues isolated from one specific hemisphere (left or right) were used to perform assays and were always matched for all the animals.

### Preparation of Tissue Homogenates for Enzyme Activity Assays

Mice brain tissues were retrieved from the −80 freezer and consecutively extracted in three different buffers which systematically isolated soluble proteins, salt-soluble ionic proteins, and detergent soluble membrane proteins, respectively. Initially, the brain tissues were homogenized (IKA-Ultra-turrax T8, Tamro AB, Sweden) with a ratio of 1:10 (tissue weight: buffer volume) in buffer-A (containing 50 mM sodium/potassium (Na/K) phosphate buffer, 2 mM EDTA, pH 7.4). The homogenates were centrifuged at 15,000 rpm at 4°C for 30 min and the supernatant was saved as soluble protein fraction. The pellet obtained was resuspended at 1:10 ratio w/v in buffer-B (buffer-A + 500 mM NaCl), homogenized and centrifuged at 15,000 rpm at 4°C for 30 min and the supernatant was saved as ionic proteins. Finally, the pellets obtained in the previous step were again resuspended at 1:10 ratio w/v in buffer-C (buffer-A + 0.6% Triton-X100) and homogenized to extract the detergent soluble membrane fractions. The final homogenate was centrifuged at 15,000 rpm at 4°C for 30 min and the supernatant was saved. All the three protein fractions were then pooled together to obtain total protein content from the brain tissue samples, which was then quantified using BCA protein assay (#23235, Pierce, Thermofisher) using bovine serum albumin (BSA) to plot the standard curve.

### Measurement of Choline Acetyltransferase Activity in Brain Tissue Homogenates

The activity of ChAT enzyme was assessed by utilizing a modified version of an integrated assay that has been used for human brain tissue homogenates as previously described (Vijayaraghavan et al., 2013). Briefly, samples were diluted 4 times with dilution buffer (10 mM Tris HCl, 1 mM EDTA, 0.05% Triton-X100, 0.1% BSA) and 10 μl of diluted samples were plated in a 384-well plate. Control wells contained 10 μl of the same samples in triplicate but were heat denatured (3 thermal cycle: 95°C for 20 s, 99°C for 8 min, 30°C for 20 s) to nullify ChAT enzyme activity and also to serve as controls for internal choline levels of the samples. Simultaneously, a standard curve of choline chloride, which was used to estimate the concentration of choline in the samples post reaction completion (with cocktail-A as mentioned below), was also included in the same 384-well plate, which contained 50 μl of each standard in triplicate starting from 100 μM and serially diluted until 0.39 μM.

When the plate set-up is ready as mentioned above, the reaction in sample wells (but not in choline chloride standard wells) was started by adding 40 μl of cocktail-A, which was prepared in dilution buffer containing acetyl coenzyme-A lithium salt 62.5 μM (ACoA; #A2181, Sigma-Aldrich), phosphotransacetylase 1.25 U/ml (#P2783, Sigma-Aldrich), lithium potassium acetyl-phosphate 14.78 mM (#01409, Sigma-Aldrich), choline chloride 37.5 μM (#C7017, Sigma-Aldrich) and eserine hemisulfate 0.075 mM (#E8625, Sigma-Aldrich). The plate was sealed and incubated in a humidified chamber for 1 h at 38°C with constant orbital shaking. Plates were then briefly put on ice for 2 min, centrifuged at 1000 rpm for 1 min and the final reaction was initiated in all wells including choline standards by adding 25 μl of cocktail-B, which was prepared in 50 mM PBS containing choline oxidase 0.93 U/ml (#C5896, Sigma-Aldrich), Streptavidin-HRP 1U/5000 μl (#43-4323, Invitrogen), 4-aminoantipyrine 3 mM (#A4382, Sigma-Aldrich), phenol 6.3 mM (#P3653, Sigma-Aldrich), pH-7.6. Absorbance of the wells was read in a spectrophotometer (Infinite M1000, Tecan) at 500 nm for 1 h kinetically at 1-min interval with 3 sec orbital shaking before every reading. ChAT activity in every sample was calculated using the formulae as described previously (Vijayaraghavan et al., 2013) and normalized to their respective protein content.

### Measurement of Cholinesterase’s (AChE and BuChE) Activity in Brain Tissue Homogenates

The acetylcholine hydrolysing activity present within the brain tissue homogenates were performed by measuring the specific individual activities of acetylcholinesterase (AChE) and butyrylcholinesterase (BuChE), respectively, by using a modified Ellman’s colorimetric method as described previously (Darreh-Shori et al., 2002). Briefly, the homogenates were diluted 8 times in dilution buffer (50 mM Na/K phosphate buffer, 2 mM EDTA, 166 mM NaCl, 0.2% Triton-X100, pH 7.4) and 50 μl of each diluted sample were plated in triplicate in 384-well plates (#464718, Nunc MaxiSorp). The reaction was started by adding 25 μl of the substrate master mix, which was different for measuring AChE and BuChE. To specifically measure AChE activity, the master mix contained 0.5 mmol/L final concentration of acetylthiocholine (ATC; Sigma, St. Louis, MO, United States) as the substrate, ethopropazine (EPP, 0.1 mmol/L final concentration) (Sigma-Aldrich) a specific inhibitor of BuChE and 0.38 mmol/L final concentration of Ellman’s reagent, 5,5′-dithio-bis(2-nitrobenzoic acid) (DTNB; Sigma-Aldrich). Similarly, to specifically measure BuChE activity, the master mix contained 1 mmol/L final concentration of butyrylthiocholine (BTC; Sigma-Aldrich) as the substrate, 0.0011 mmol/L final concentration of BW284c51 as specific AChE inhibitor and 0.38 mmol/L final concentration of DTNB. The reaction was allowed to run for 20 min and the absorbance was read in a spectrophotometer (Infinite M1000, Tecan) continuously at 1-min intervals with 3 s orbital shaking before every reading at 405 nm in a kinetic mode. Rate of reaction was extracted and utilized to calculate AChE and BuChE activity using the extinction coefficient of DTNB (1.36 × 10^4^/M/cm). The activity of AChE and BuChE were normalized to the protein content of the individual samples.

### Determination of NGF Protein Levels in Brain Homogenates

Total NGF protein levels in the brain homogenates were measured using ELISA kit (#DY256, R&D Systems), according to the manufacturer’s recommendation with minor modifications. Mouse β-NGF (#1156-NG-100, R&D Systems) was used to prepare a standard curve to quantify the NGF levels from the brain tissue homogenate samples. Briefly, 384-well plates were coated with 50 μl of capture antibody (2 μg/ml, in carbonate buffer, pH 9.8) at 4°C overnight. Plates were then decanted, washed 1 × 5 min with TBS and blocked with 5% BSA in a carbonate buffer for 1 h at room temperature (RT). Following 3 × 5 min washes with TBST^0.05%^, 50 μl of samples (four times diluted) and standards (S1 = 4 ng, serially double diluted until S10) diluted in reagent diluent (1% BSA in PBS, 0.01% sodium azide, 0.22 μm filtered, pH 7.4) were plated in respective wells and incubated overnight at 4°C. Subsequently, the plates were washed 3 × 5 min with TBST^0.05%^ and incubated with 50 μl of biotinylated capturing antibody (50 ng/ml in reagent diluent) for 3 h at RT. Following 3 × 5 min washes with TBST^0.05%^, the wells were further incubated for 2 h at RT with 50 μl of streptavidin conjugated alkaline phosphatase (AP; 1:10000, diluted in reagent diluent; #11093266910, Roche Diagnostics). Finally, the wells were washed 2 × 5 min with TBST^0.05%^ followed by 1 × 5 min with diethanolamine buffer (DEA; 1.0 M, pH 9.8). 50 μl of AP-substrate (1 mg/ml p-nitrophenyl-Na2-6H_2_O in DEA buffer) was added and the change in absorbance was read every 5-min for 13 cycles (total reading time 1 h) kinetically at 405 nm in a spectrophotometer (Infinite M1000, Tecan). Rate of reaction data was extracted and used to quantitate NGF levels in tissue homogenate samples using 4-parametric standard curve.

### RNA Isolation and Quantitative PCR

Total RNA was isolated from frozen tissues using Trizol Reagent (Thermo Fisher Scientific) according to the manufacturer’s protocol and RNA quantity and quality (absorbance 260/280 nm > 1.8) were assessed using a NanoDrop (NanoDrop Technologies, LLC, Wilmington, DE, United States). 200 ng of DNase I (Thermo Fisher Scientific, MA, United States)-treated total RNA was reverse transcribed to complementary DNA using random hexamer primers and RevertAid Reverse Transcriptase (Thermo Fisher Scientific, MA, United States). Complementary DNA was diluted 1:10 and stored at −20°C until analysis. Quantitative PCR was performed with BioRad C1000 Touch Thermal Cycler upgraded to CFX384 System (BioRad), supplied with SYBR Green I Master (Roche) and 250 pmol primers, in 10 μl total volume in 384-well plates. Each sample was run in triplicate. Data were normalized to the geometric mean of *Actb* and *Gapdh* housekeeping genes expression. Results for a biological repeat were discarded when the Cq value for one or more of the replicates was 40 or 0, or when the Cq difference between replicates was >1. Primer sequences are provided in Supplementary Table 1.

### Y-Maze Test

The Y-maze test measures the ability of mice to recognize the environment they have already explored, and it is therefore a commonly used task to assess spatial recognition memory (Kraeuter et al., 2019). The maze consists of three arms with Plexiglas walls (30 cm × 8 cm × 15 cm), and with an angle of 120° between each arm. During the experiment, the mice were introduced at the center of the maze and allowed to freely explore the maze. The total number of entries into each arm (A, B, C) was measured over a period of 10 min. All the trials were recorded using the Ethovision XT 13 tracking system (Noldus et al., 2001). The arms were cleaned with a 70% ethanol solution before the experiment started and with water between each animal. Spontaneous alternation is the subsequent entry into a novel arm over the course of three entries, and the % of spontaneous alternations was calculated by the number of actual alternations/(total arm entries − 2) × 100 (Ghosal et al., 2009).

### Statistical Analysis

**FIGURE 1:**
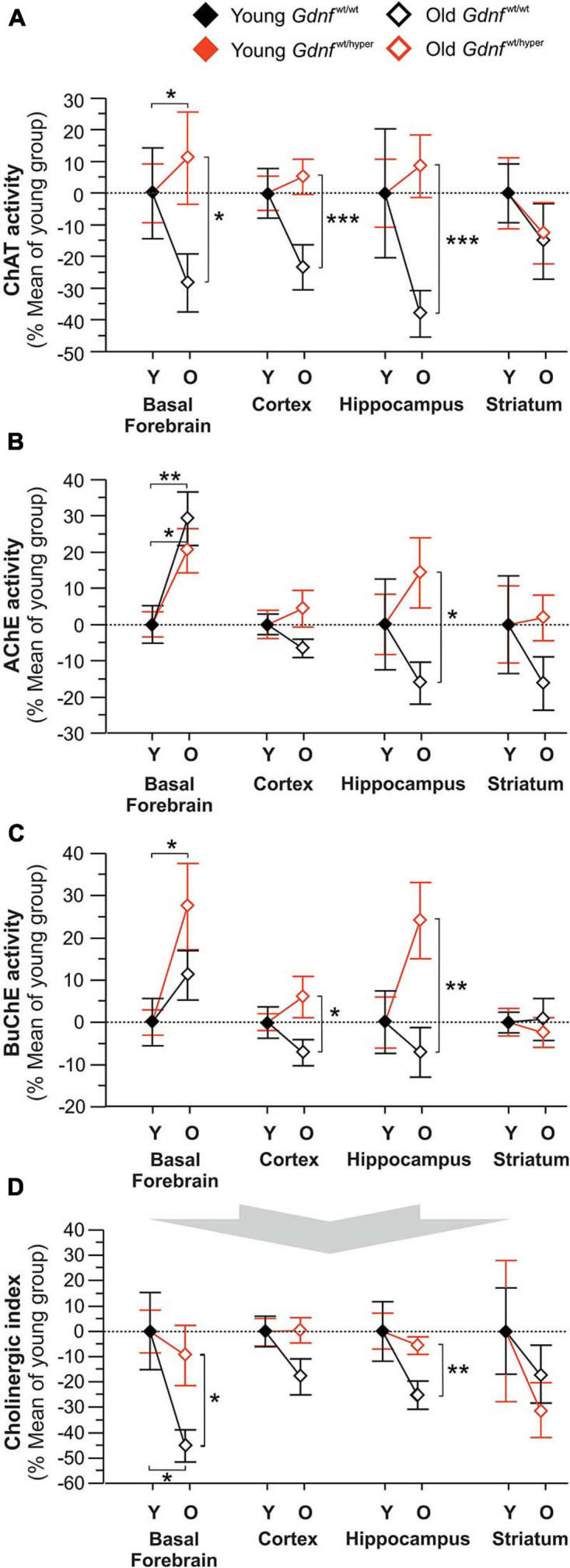
Changes in the key cholinergic enzymes in young and old *Gdnf*^wt/wt^ and *Gdnf*^wt/hyper^ mice. The activity of the acetylcholine-biosynthesizing enzyme, ChAT **(A)** and acetylcholine-degrading enzymes, AChE **(B)** and BuChE **(C)** measured in different brain regions of young (Y) and old (O) *Gdnf*^wt/wt^ and *Gdnf*^wt/hyper^ mice. The data are given as percent of mean values in young animals. The raw data is provided in the Supplementary File. **(D)** The corresponding changes in Cholinergic index in the *Gdnf*^wt/hyper^ mice and wild-type littermates. The Cholinergic index provides a measure between ACh biosynthesis by ChAT relative ACh-biodegradation by cholinesterases (AChE and BuChE). This index is calculated as the ratio between ChAT activity to the ChE activity. *n* = 10 per group (young) and *n* = 8 per group (old). Data are presented as mean ± SEM. *p < 0.05; **p < 0.01; ***p < 0.001. AChE, acetylcholinesterase; BuChE, butyrylcholinesterase; ChAT, choline acetyltransferase.

**FIGURE 2:**
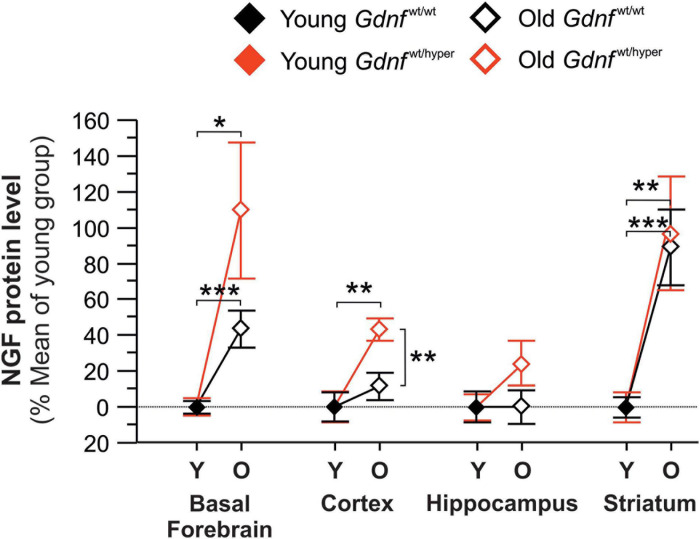
Changes in the protein levels of NGF in the brain of young and old *Gdnf*^wt/wt^ and *Gdnf*^wt/hyper^ mice. NGF protein level was measured in the basal forebrain, cortex, hippocampus, and striatum of young (Y) and old (O) *Gdnf*^wt/hyper^ and *Gdnf*^wt/wt^ mice. The data are given as percent of mean values in young animals. The raw data is provided in the Supplementary File. *n* = 10 per group (young) and *n* = 8 per group (old). Data are presented as mean ± SEM. *p < 0.05; **p < 0.01; ***p < 0.001.

**FIGURE 3:**
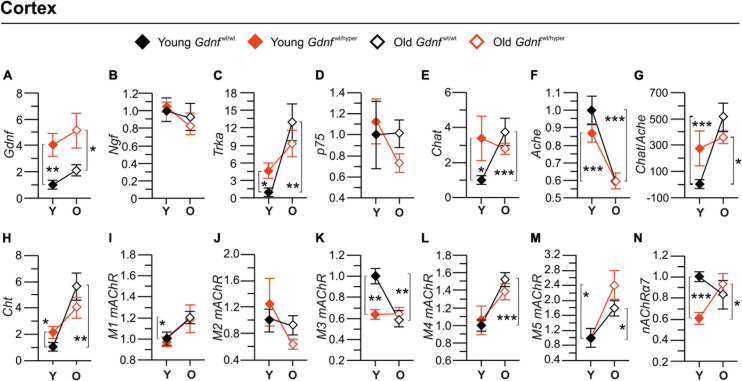
Gene expression of the main cholinergic genes in the cortex. Graphs **(A–F,H–N)** show the fold-changes in the gene expressions relative to the corresponding gene expression level in the cortex of the young *Gdnf*^wt/wt^ mice. The gene expressions were measured by quantitative PCR. Graph **(G)** shows percent changes in a *Cholinergic index* calculated based on the ratio between the gene expressions of *Chat* to *Ache*. *n* = 6 (young *Gdnf*^wt/wt^), *n* = 5 (old *Gdnf*^wt/wt^), *n* = 7 (young *Gdnf*^wt/hyper^), *n* = 6 (old *Gdnf*^wt/hyper^). Data are presented as mean fold changes ± SEM. *p < 0.05; **p < 0.01; ***p < 0.001. *Ache*, acetylcholinesterase; *Chat*, choline acetyltransferase; *Cht*, choline transporter; *Gdnf*, glial cell line-derived neurotrophic factor; *M1* mAChR, m1 muscarinic ACh receptor; *M2*, m2 muscarinic ACh receptor; *M3* mAChR, m3 muscarinic ACh receptor; *M4*, m4 muscarinic ACh receptor; *M5*, m5 muscarinic ACh receptor; *p75*, p75 neurotrophin receptor; α*7-nAChR*, alpha 7 nicotinic ACh receptor; *Ngf*, nerve growth factor; O, old; *Trka*, tropomyosin receptor kinase A; Y, young.

**FIGURE 4:**
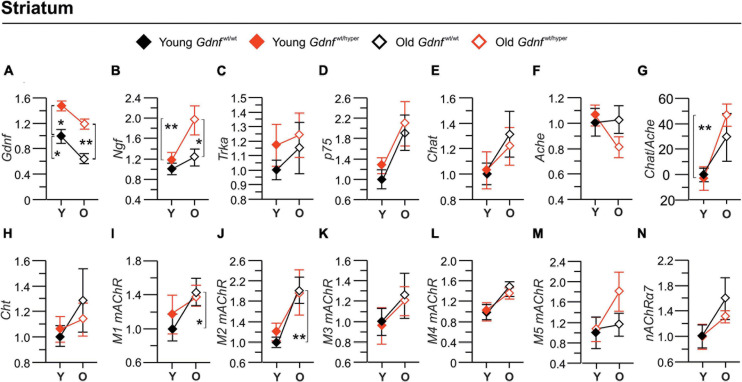
Gene expression of the main cholinergic genes in striatum. Graphs **(A–F,H–N)** show the fold-changes in the gene expressions relative to the corresponding gene expression level in the striatum of the young *Gdnf*^wt/wt^ mice. The gene expressions were measured by quantitative PCR. In panel **(G)** is shown percent changes in a *Cholinergic index* calculated based on the ratio between the gene expressions of *Chat* to *Ache*. *n* = 5 (young *Gdnf*^wt/wt^), *n* = 6 (old *Gdnf*^wt/wt^), *n* = 6 (young *Gdnf*^wt/hyper^), *n* = 7 (old *Gdnf*^wt/hyper^). Data are presented as mean folds-changes ± SEM. *p < 0.05; **p < 0.01. *Ache*, acetylcholinesterase; *Chat*, choline acetyltransferase; *Cht*, choline transporter; *Gdnf*, glial cell line-derived neurotrophic factor; *M1* mAChR, m1 muscarinic ACh receptor; *M2*, m2 muscarinic ACh receptor; *M3* mAChR, m3 muscarinic ACh receptor; *M4*, m4 muscarinic ACh receptor; *M5*, m5 muscarinic ACh receptor; *p75*, p75 neurotrophin receptor; α*7-nAChR*, alpha 7 nicotinic ACh receptor; *Ngf*, nerve growth factor; O, old; *Trka*, tropomyosin receptor kinase A; Y, young.

**FIGURE 5:**
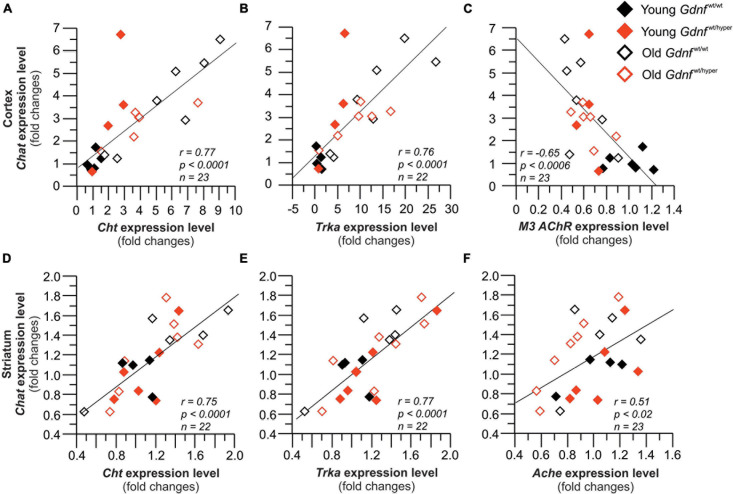
Gene expression of the key cholinergic enzyme, *Chat* strongly correlates with the expression of the related genes. Graphs **(A–C)** show correlations in the cortex, and **(D–F)** show the correlations in the striatum. Cortex: *n* = 6 (young *Gdnf*^wt/wt^), *n* = 5 (old *Gdnf*^wt/wt^), *n* = 7 (young *Gdnf*^wt/hyper^), *n* = 6 (old *Gdnf*^wt/hyper^). Striatum: *n* = 5 (young *Gdnf*^wt/wt^), *n* = 6 (old *Gdnf*^wt/wt^), *n* = 6 (young *Gdnf*^wt/hyper^), *n* = 7 (old *Gdnf*^wt/hyper^). *Ache*, acetylcholinesterase; *Chat*, choline acetyltransferase; *Cht*, choline transporter; *M3 mAChR*, m3 muscarinic ACh receptor 3; *Trka*, tropomyosin receptor kinase A.

**FIGURE 6:**
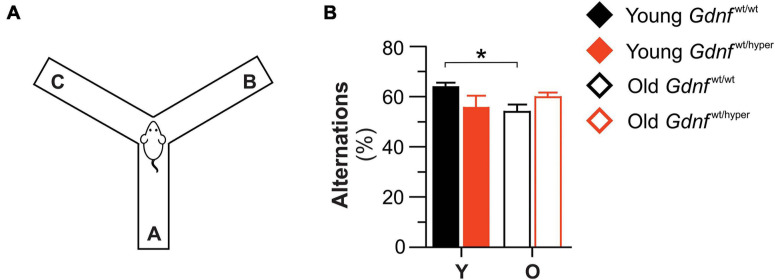
*Gdnf*^wt/hyper^ mice resist age-related decline in Y-maze test. **(A)** The spatial recognition memory of *Gdnf*^wt/wt^ and *Gdnf*^wt/hyper^ mice was assessed with the Y-maze test. **(B)** Upon aging, the number of alternations was significantly decreased in *Gdnf*^wt/wt^ mice, but not in *Gdnf*^wt/hyper^ animals. *n* = 7 (young *Gdnf*^wt/wt^), *n* = 5 (old *Gdnf*^wt/wt^), *n* = 6 (young *Gdnf*^wt/hyper^), *n* = 12 (old *Gdnf*^wt/hyper^). Data are presented as mean ± SEM. *p < 0.05. O, old; Y, young.

For biochemical assessments of cholinergic enzymes activity and NGF protein analysis (Figures 1, 2) data are presented as the percent of the mean of the young animals since we did not observe major significant differences among young mice. Therefore, only differences in old animals between the genotype groups were considered and assessed by unpaired *t*-test. For gene expression analysis (Figures 3, 4), data are presented as mean fold change compared to young *Gdnf*^wt/wt^ animals. Differences between groups were assessed by unpaired *t*-test. Spearman correlation analysis was used to analyze possible associations between variables and the results were then visualized using simple linear regression graphs (Figure 5). Results from the Y-maze test (Figure 6) are presented as mean of each group, and comparisons were assessed with one-way Analysis of Variance (ANOVA) test. Significant levels were set at *p* < 0.05. The non parametric Mann-Whitney *U* test was used to assure that significant results were not caused by the effect of outliers.

## Results

### Increased Expression of Endogenous GDNF Induces Resilience Against an Age-Dependent Decline in Cholinergic System

The activities of the key cholinergic enzymes ChAT, AChE, and BuChE were analyzed at different ages (young and old) in *Gdnf*^wt/wt^ and *Gdnf*^wt/hyper^ mice in various brain regions. ChAT activity did not statistically differ between young *Gdnf*^wt/hyper^ and *Gdnf*^wt/wt^ littermates in any of the brain regions that were assessed (Supplementary Table 2). We found a statistically significant age-dependent decline in ChAT activity in *Gdnf*^wt/wt^ animals (∼40% in basal forebrain, ∼30% in cortex, ∼45% in hippocampus) compared to *Gdnf*^wt/hyper^ mice (Figure 1A). In contrast, no significant age-dependent changes in the activity of ChAT were found in striatum in both groups of animals (Figure 1A).

Like ChAT, no statistically significant differences in the activity of ACh degrading enzyme, AChE, were observed between young *Gdnf*^wt/hyper^ and *Gdnf*^wt/wt^ littermates in any of the brain regions that were assessed (Supplementary Table 3). However, a comparison between the young and old animals revealed that the activity of AChE was significantly increased in the basal forebrain by about 20% in old *Gdnf*^wt/hyper^ and by about 30% in old *Gdnf*^wt/wt^ littermates (Figure 1B). In contrast, no age-dependent changes in the activity of AChE were observed among the *Gdnf*^wt/hyper^ and the *Gdnf*^wt/wt^ littermates in cortex and striatum. In the hippocampus, the old *Gdnf*^wt/hyper^ exhibited about 30% higher AChE activity compared to the old *Gdnf*^wt/wt^ littermates (Figure 1B).

Current data suggests that BuChE cooperates with AChE in degrading ACh (Nordberg et al., 2013). Therefore, we analyzed the activity of BuChE in the same brain regions and found that BuChE showed a similar pattern of relative changes to AChE activity among the animal groups (Figure 1C). The activity of BuChE was on average about 30-fold lower than the activity of AChE regardless of genotype or age of the animals (Supplementary Table 4).

Free choline is one of the two substrates required for biosynthesis of acetylcholine. However, the levels of choline neither showed genotype-dependent nor age-dependent significant changes among the animal groups (Supplementary Table 5). We observed that choline levels were about 4-fold higher in striatum than in the other brain regions (Supplementary Table 5).

Normal cholinergic metabolism involves a balance between ACh production, release, and degradation by AChE and BuChE. *Cholinergic index*, calculated as ratio of ChAT activity and sum of AChE and BuChE activities in human cerebrospinal fluid provides clinically relevant information on cholinergic status in patients with AD (Karami et al., 2019). We performed the similar calculations on our data from mouse models used in this study. Old *Gdnf*^wt/wt^ mice exhibited significantly lower *Cholinergic index* in basal forebrain (about 35%) and hippocampus (about 20%) compared to the old *Gdnf*^wt/hyper^ mice, but not in the cortex and the striatum (Figure 1D).

### Increase in Endogenous GDNF Expression Induces an Increase in NGF Protein Expression Upon Aging

Next, we measured total NGF protein content in the same brain regions where the activity of the cholinergic enzymes was assessed. We found that the levels of total NGF protein were similar between genotypes in young mice (Supplementary Table 6). Upon aging, NGF levels were significantly increased in the basal forebrain and striatum in both *Gdnf*^wt/wt^ and *Gdnf*^wt/hyper^ groups (110 vs. 44% in basal forebrain and 97 vs. 84% in striatum compared to the young group) (Figure 2). In the cortex, the level of NGF protein significantly increased upon aging in *Gdnf*^wt/hyper^ mice, but not in *Gdnf*^wt/wt^ littermates (40 vs. 10%) (Figure 2). We did not observe significant changes in NGF level in the hippocampus in both genotype upon aging (2 vs. 0.1%) (Figure 2).

In conclusion, we observed an overall increase in NGF protein levels upon aging. Genotype specific alterations were observed in the cortex, where old *Gdnf*^wt/hyper^ mice displayed increased NGF levels relative to the wild type controls. Among the different forms of NGF, proNGF was the predominant form observed in cortical brain tissue homogenates (Supplementary Figure 1).

### Gene Expression Analyses in the Cortex and Striatum of *Gdnf*^wt/wt^ and *Gdnf*^wt/hyper^ Mice

To further evaluate the observed genotype- and age-dependent differential changes in the cholinergic system, we measured the expression level of genes involved in the maintenance and function of the cholinergic system.

As a positive control, we measured mRNA encoding for GDNF levels and, as reported previously, we observed almost 2–3-fold upregulation in *Gdnf*^wt/hyper^ mice relative to the wild type controls in the cortex (Figure 3A) and striatum (Figure 4A). In the cortex, the gene expression level of *Ngf* did not differ between genotypes upon aging (Figure 3B), contrary to the protein expression levels which was significantly different among old *Gdnf*^wt/wt^ and *Gdnf*^wt/hyper^ groups (Figure 2). The level of the NGF receptor, *Trka*, was significantly increased in young *Gdnf*^wt/hyper^ mice, and showed a significant age-dependent increase in *Gdnf*^wt/wt^ mice (Figure 3C) but not in the *Gdnf*^wt/hyper^ mice. On the other hand, in the striatum, *Ngf* gene expression significantly increased in an age-dependent manner in *Gdnf*^wt/hyper^ mice (Figure 4B) similar to increased protein levels observed in both the genotypes, whereas *Trka* expression did not show age-related changes between the genotypes (Figure 4C). We did not observe significant changes in the expression level of *p75* neurotrophin receptor both in the cortex (Figure 3D) and in the striatum (Figure 4D).

Next, we analyzed the expression level of cholinergic enzymes, *Chat* and *Ache*, as well as the choline reuptake transporter (*Cht*). We found that *Chat* expression was significantly higher in young *Gdnf*^wt/hyper^ mice compared to the control littermates in the cortex (Figure 3E), but not in the striatum (Figure 4E). In addition, the *Chat* levels showed a significant age-dependent increase in the cortex (Figure 3E), but not in the striatum (Figure 4E), in the *Gdnf*^wt/wt^ group. We found a significant age-dependent decline of *Ache* expression in both groups in the cortex (Figure 3F), but no changes were observed in striatum (Figure 4F). It is of note that we observed no correlation between mRNA and enzyme activity levels of *Chat* and *Ache* (shown in Figure 1) in both cortex and striatum in young and old animals, suggesting a compensatory response or other regulatory mechanisms of these enzymes. Next, we calculated a gene-based *Cholinergic index* as the relative gene expression of the two main cholinergic genes i.e., *Chat* and *Ache* both in the cortex (Figure 3G) and in the striatum (Figure 4G). In the cortex, the gene-based *Cholinergic index* significantly increased upon aging in *Gdnf*^wt/wt^, but not in the *Gdnf*^wt/hyper^ mice, whereas in the striatum a significant increase in this index was observed in *Gdnf*^wt/hyper^ mice. Similar to *Chat*, the *Cht* expression in cortex was about twofold higher in young *Gdnf*^wt/hyper^ mice compared to the young *Gdnf*^wt/wt^ littermates (Figure 3H) and several folds increased in the *Gdnf*^wt/wt^ mice upon aging (Figure 3H). These changes were not observed in the striatum (Figure 4H). Finally, we measured the expression level of several cholinergic receptors. In the cortex, we found an increase in the level of *M1* muscarinic ACh receptor, but not *M2*, in *Gdnf*^wt/wt^ mice upon aging (Figures 3I,J), whereas in the striatum both *M1* and *M2* expression significantly increased in old *Gdnf*^wt/wt^ mice (Figures 4I,J). We observed a differential age-related and genotype dependent gene expressions in *M3*, *M4*, and *M5* muscarinic ACh receptors and the α7 subtype of the nicotinic ACh receptors (Figures 3K–N) in the cortex, but not in the striatum (Figures 4K–N).

In conclusion, we observed primarily age-related changes in the expression level of key genes involved in cholinergic transmission in the cortex of *Gdnf*^wt/wt^ animals compared to the *Gdnf*^wt/hyper^ mice. In the striatum, we observed an increase in the expression level of *Ngf* in *Gdnf*^wt/hyper^ mice and *M1* and *M2* in *Gdnf*^wt/wt^ mice upon aging, whereas other genes did not show significant changes between genotype and age groups.

### Expression of ChAT Correlates With Other Cholinergic Genes in the Cortex and Striatum

To gain more insight into the interrelationship of cholinergic gene expressions, we looked at the correlation pattern of *Chat* - the key enzyme responsible for the biosynthesis of ACh – and other cholinergic genes in the cortex and in the striatum. In the cortex, *Chat* gene expression strongly correlated with the expression of *Cht*, *Trka* and the muscarinic receptor *M3* (Figures 5A–C). In the striatum, *Chat* gene expression strongly correlated with the gene expression of *Cht*, *Trka* and *Ache* (Figures 5D–F). This data shows a strong correlation between the expression levels of the key enzyme in ACh biosynthesis, i.e., *Chat*, the key players in cholinergic transmission, i.e., choline transporter *Cht*, and the NGF receptor *Trka* in the cortex and striatum of *Gdnf*^wt/wt^ and *Gdnf*^wt/hyper^ mice upon aging.

### *Gdnf*^wt/wt^ but Not *Gdnf*^wt/hyper^ Mice Show Age-Dependent Decline in Spatial Working Memory in Y-Maze Test

The Y-maze assesses short term spatial working memory in mice. Spontaneous alternation between the three arms of the Y-maze can be assessed by allowing mice to explore all three arms of the maze. This behavior is driven by an innate curiosity of rodents to explore previously unvisited areas. A mouse with intact working memory, and hence intact prefrontal cortical functions, will remember the arms previously visited and show a tendency to enter a less recently visited arm (Kraeuter et al., 2019). During the test, the mouse was placed at the center of the maze and the total number of entries into each arm (A, B, C) was measured over a period of 10 min (Figure 6A). We found no difference between genotypes in young and aged mice (Figure 6B). However, while *Gdnf*^wt/wt^ mice exhibited significantly fewer spontaneous alternations upon aging, old *Gdnf*^wt/hyper^ mice performed as well as the young animals (Figure 6B). These data show that *Gdnf*^wt/hyper^ mice do not undergo an age-related decline in cognitive function, as assessed by the Y-maze test, observed in the wild-type littermates.

## Discussion

In this study, we investigated whether an approximate 2–3-fold increase in endogenous GDNF (a well-known dopaminergic trophic factor) expression influences brain cholinergic system function in young and old mice. We found that, compared to the wild type controls, *Gdnf*^wt/hyper^ mice display a reduced decline in cholinergic function upon normal aging. Our results suggest that GDNF may influence cholinergic transmission by regulating activity of key components and regulators of the cholinergic machinery, including ACh-biosynthesizing enzyme ChAT, the high affinity choline transporter (ChT) and NGF, respectively. Intriguingly, this effect seems to be significant to the cholinergic system in the cortex, which plays a key role in cognition (Miller, 2000) and it is mostly affected in several conditions associated with dementia, including AD, PDD, and LBD (Francis and Perry, 2007; Dickerson et al., 2009). Changes in the activity and gene expression of the components of the cholinergic machinery in the striatum, which is mainly involved in controlling motor function and reward (Lanciego et al., 2012; Cox and Witten, 2019), were relatively minor. In addition, upon aging we found an increase in NGF protein levels in the cortex of old *Gdnf*^wt/hyper^ mice that was absent in the control group. We observed that proNGF is the form of NGF which was increased in cortical tissues of old animals, supporting previous observations that proNGF is the predominant form of NGF present in the brain (Fahnestock et al., 2001). Several studies have shown the ability of NGF to support the maintenance and survival of the cholinergic neuronal system (Allen et al., 2013) and to contribute to the survival and regeneration of cholinergic neurons in age-related diseases, such as AD (Mitra et al., 2019), suggesting that an increase in NGF in the brain of *Gdnf*^wt/hyper^ mice may contribute to the preserved cholinergic function upon aging.

In the cortex, gene expression of key components of the cholinergic system, including *Chat*, *Ache*, and *Cht*, as well as several downstream components of cholinergic pathway, were differentially regulated in *Gdnf*^wt/hyper^ compared to *Gdnf*^wt/wt^ mice. Interestingly, increased endogenous GDNF expression did not alter gene expression and/or activity of the cholinergic enzymes in the striatum, the brain region where GDNF is predominantly expressed (Hidalgo-Figueroa et al., 2012), but showed increased *Ngf* gene expression when compared to the cortex. The striatum is the brain area where the cholinergic interneurons are located (Gonzales and Smith, 2015) and has the highest concentration of cholinergic markers in the brain (Benarroch, 2012). Striatal cholinergic interneurons are spared in AD and in related dementia disorders, such as LBD and PDD which are known to cause early degeneration of the central cholinergic neurons in the basal forebrain (Kotagal et al., 2012). Thus, the downstream effect of endogenous GDNF overexpression seems to be directed to the cholinergic neurons in the cortex, which are more vulnerable to deterioration with advancing age and in dementia disorders. However, it is currently unclear how GDNF exerts this effect and future investigation is warranted.

Interestingly, we also observed a peculiar result of a divergence between enzyme activity-based *Cholinergic index*, which was almost universally decreased in *Gdnf*^wt/wt^ mice upon aging, and the gene-based *Cholinergic index*, which was increased upon aging in the cortex and striatum. This may indicate a compensatory response in the brain of wild type mice to prevent the age-dependent decline in cholinergic function through gene regulation.

Aging induces a gradual decline in cognitive function. Decline in Y-maze performance has been previously reported upon normal aging (Li et al., 2020) and in several mouse models of AD (Webster et al., 2014). Similarly, we observed a decline in Y-maze performance in old *Gdnf*^wt/wt^ animals but interestingly not in old *Gdnf*^wt/hyper^ mice. We recently found that young *Gdnf*^wt/hyper^ mice have mildly impaired long-term memory in the Morris water maze test, accompanied by an increased number of PV^+^ neurons and inhibitory tone in the hippocampus (Marshall et al., 2021). However, Morris water maze test with old animals did not reveal differences between *Gdnf*^wt/hyper^ mice and wild-type controls (Turconi et al., 2020), suggesting a complex adaptation response that requires time to be established. Since age-related reduction in *Cholinergic index*, indicating a net reduction in ACh’s availability in the system, was restricted to the wild type animals, GDNF levels either directly or indirectly may regulate this response. In addition to ACh, other neurotransmitters, including dopamine and serotonin, are known to be involved in regulating cognitive processes (Nieoullon, 2002; Xu et al., 2012; Svob Strac et al., 2016). Therefore, based on current data we cannot exclude the possibility that other than cholinergic neurotransmitter systems may affect cognitive performance in *Gdnf*^wt/hyper^ mice upon aging.

How does GDNF signaling influence cholinergic network function upon aging? One intriguing possibility is *via* the enhanced dopaminergic function in *Gdnf*^wt/hyper^ mice (Kumar et al., 2015; Turconi et al., 2020). There is a well-established close crosstalk between dopaminergic and cholinergic pathways in the brain, for instance in modulating cognitive function (Levin et al., 1990) and addiction (Tan and Bullock, 2008; Lester et al., 2010; Lewis et al., 2020). In the striatum, these pathways interact through dopaminergic receptors D1 and D2, expressed on cholinergic interneurons, and muscarinic and nicotinic ACh receptors, expressed on dopaminergic axons (Imperato et al., 1993; DeBoer et al., 1996; Smolders et al., 1997; Kaiser and Wonnacott, 2000; Zhang et al., 2002; Zhou et al., 2002; Quik and McIntosh, 2006). Notably, in the cortex, an increase in endogenous GDNF levels was associated with a 2–3-fold higher expression of the two main cholinergic genes involved in the biosynthesis of ACh i.e., *Chat* and *Cht* in young *Gdnf*^wt/hyper^ mice. Therefore, we cannot rule out the possibility that ACh turnover is increased in young *Gdnf*^wt/hyper^ mice, which may partially explain why the cholinergic system is more resilient to an age-dependent decline in the *Gdnf*^wt/hyper^ mice.

Our current study is first of its kind but bares several limitations. First, our pilot study is purely explorative and currently we are unable to provide a mechanistic explanation, inviting the need for future research. Second, gene expression and correlation analysis were limited to two brain areas i.e., cortex and striatum, because of samples data availability and large size of the dataset required for a reliable multiple correlation analysis. Moreover, given that GDNF is ubiquitously and constitutively expressed in *Gdnf*^wt/hyper^ mice, future studies should be designed to address the outcome of adult onset increase of endogenous GDNF on cholinergic transmission, including the analysis of conditional GDNF hypermorphic mice (Mätlik et al., 2019).

In conclusion, we show that long-term increases in endogenous GDNF expression protects against age-related decline in key players of the cholinergic transmission and function. Our work identifies endogenous GDNF as a modulator of the cholinergic system and encourages future studies on GDNF in conditions characterized by age-dependent progressive deterioration in cholinergic function, such as AD, LBD, and PDD.

## Data Availability Statement

The raw data supporting the conclusions of this article will be made available by the authors, without undue reservation.

## Ethics Statement

The animal study was reviewed and approved by the national Animal Experiment Board of Finland (license numbers ESAVI/11198/04.10.07/2014 and ESAVI/12046/04.10.07/2017). All animal experiments were conducted according to the 3R principles of the European Union Directive 2010/63/EU governing the care and use of experimental animals, following local laws and regulations [Finnish Act on the Protection of Animals Used for Scientific or Educational Purposes (497/2013), Government Decree on the Protection of Animals Used for Scientific or Educational Purposes (564/2013)].

## Author Contributions

SM, GT, and MA performed the experiments. GT and KM dissected the brain tissues. SM, GT, and TD-S analyzed the data and wrote the manuscript. TD-S and GT prepared the figures. ME, J-OA, and TD-S supervised the project, planned experiments, critically reviewed the manuscript, and provided funding. J-OA and TD-S conceptualized the study. All authors have read and approved the manuscript.

## Conflict of Interest

The authors declare that the research was conducted in the absence of any commercial or financial relationships that could be construed as a potential conflict of interest.

## Publisher’s Note

All claims expressed in this article are solely those of the authors and do not necessarily represent those of their affiliated organizations, or those of the publisher, the editors and the reviewers. Any product that may be evaluated in this article, or claim that may be made by its manufacturer, is not guaranteed or endorsed by the publisher.
