# Supplementary material for: Increased Endogenous GDNF in Mice Protects Against Age-Related Decline in Neuronal Cholinergic Markers
Source: Front Aging Neurosci. 2021 Aug 12;13:714186. doi: 10.3389/fnagi.2021.714186 (PMC8406776; doi:10.3389/fnagi.2021.714186)
Supplement: Supplementary file 1 [file Data_Sheet_1.docx]

**Increased endogenous GDNF in mice protects against age-related decline in neuronal cholinergic markers**

Sumonto Mitra ^1^*^†^, Giorgio Turconi ^2†^, Taher Darreh-Shori ^1^, Kärt Mätlik ^2^, Matilde Aquilino ^2^, Maria Eriksdotter ^1, 3^, Jaan-Olle Andressoo ^1, 4^

**Author affiliations**

^1^ Division of Clinical Geriatrics, Center for Alzheimer Research, Department of Neurobiology, Care Sciences and Society (NVS), Karolinska Institutet, Huddinge, Sweden.

^2^ Department of Pharmacology, Faculty of Medicine & Helsinki Institute of Life Science, University of Helsinki, Helsinki, Finland.

^3^ Theme Inflammation and Aging, Karolinska University Hospital, Huddinge, Sweden.

^4^ Division of Neurogeriatrics, Center for Alzheimer Research, Department of Neurobiology, Care Sciences and Society (NVS), Karolinska Institutet, Stockholm, Sweden.

*Corresponding author : Sumonto Mitra, Division of Clinical Geriatrics, Center for Alzheimer Research, Department of Neurobiology, Care Sciences and Society (NVS), Karolinska Institutet, 14183 Huddinge, Sweden. Email – [sumonto.mitra@ki.se](mailto:sumonto.mitra@ki.se); Phone – +46-734967524

^†^ These authors share first authorship

**Keywords:** Glial cell-line derived neurotrophic factor (GDNF), Nerve growth factor (NGF), Aging, Cholinergic markers, Cholinergic index, Choline acetyltransferase (ChAT), Acetylcholinesterase (AChE), Brain

**Running title:** GDNF prevents age-related cholinergic decline

# SUPPLEMENTARY MATERIALS

**Supplementary Table 1.** List of primers used for quantitative PCR.

**Supplementary Table 2.** Descriptive data of choline acetyltransferase (ChAT) activity in the basal forebrain, cortex, hippocampus, and striatum of *Gdnf*^wt/wt^ and *Gdnf*^wt/hyper^ mice.

**Supplementary Table 3.** Descriptive data of acetylcholine esterase (AChE) activity in the basal forebrain, cortex, hippocampus, and striatum of *Gdnf*^wt/wt^ and *Gdnf*^wt/hyper^ mice.

**Supplementary Table 4.** Descriptive data of butyrylcholine esterase (BuChE) activity in the basal forebrain, cortex, hippocampus, and striatum of *Gdnf*^wt/wt^ and *Gdnf*^wt/hyper^ mice.

**Supplementary Table 5.** Descriptive data of choline level in the basal forebrain, cortex, hippocampus, and striatum of *Gdnf*^wt/wt^ and *Gdnf*^wt/hyper^ mice.

**Supplementary Table 6.** Descriptive data of nerve growth factor (NGF) level in the basal forebrain, cortex, hippocampus, and striatum of *Gdnf*^wt/wt^ and *Gdnf*^wt/hyper^ mice.

**Supplementary Figure 1.** NGF levels in cortical tissues of *Gdnf*^wt/wt^ and *Gdnf*^wt/hyper^ mice.

**Supplementary Table 1. List of primers used for quantitative PCR.**

| *Gdnf* | Forward | CGCTGACCAGTGACTCCAATATGC |
| --- | --- | --- |
|  | Reverse | TGCCGCTTGTTTATCTGGTGACC |
| *Ngf* | Forward | ACACTCTGATCACTGCGTTTTTG |
|  | Reverse | CCTTCTGGGACATTGCTATCTGT |
| *Trka* | Forward | AGAGTGGCCTCCGCTTTGT |
|  | Reverse | CGCATTGGAGGACAGATTCA |
| *p75* | Forward | GTAGCCTGCCCCTGACCAA |
|  | Reverse | GCCTCGTGGGTAAAGGAGTCT |
| *Chat* | Forward | AAGCTTCCACGCCACTTTC |
|  | Reverse | AGAGCCTCCGACGAAGTTG |
| *Ache* | Forward | TTAGGGCTGGGATATAATACGAC |
|  | Reverse | GCCCCTAGTGGGAGGAAGT |
| *Cht* | Forward | ATGTCTTTCCACGTAGAAGGACT |
|  | Reverse | TTGCCGCTGTTTTTGGTTTTC |
| *m1* | Forward | TCCCTCACATCCTCCGAAGGTG |
|  | Reverse | CTTTCTTGGTGGGCCTCTTGACTG |
| *m2* | Forward | CTGGAGCACAACAAGATCCAGAAT |
|  | Reverse | CCCCCTGAACGCAGTTTTCAGT |
| *m3* | Forward | GCAAGACCTCTGACACCAACT |
|  | Reverse | AGCAAACCTCTTAGCCAGCG |
| *m4* | Forward | CGGCTACTGGCTCTGCTACGTCAA |
|  | Reverse | CTGTGCCGATGTTCCGATACTGG |
| *m5* | Forward | TAGCATGGCTGGTCTCCTTCA |
|  | Reverse | CGCTTCCCGACCAAGTACTG |
| *nAChRα7* | Forward | GGTCATTTGCCCACTCTG |
|  | Reverse | GACAGCCTATCGGGTGAG |
| *Gapdh* | Forward | CCTCGTCCCGTAGACAAAA |
|  | Reverse | ATGAAGGGGTCGTTGATGGC |
| *Actb* | Forward | CTGTCGAGTCGCGTCCA |
|  | Reverse | ACGATGGAGGGGAATACAGC |

**Supplementary Table 2. Descriptive data of choline acetyltransferase (ChAT) activity in the basal forebrain, cortex, hippocampus, and striatum of *Gdnf*^wt/wt^ and *Gdnf*^wt/hyper^ mice.**

| **ChAT activity (pmol/min/mg)** | | | | | |
| --- | --- | --- | --- | --- | --- |
| **Brain area** | **Genotype group** | **Age group** | **Mean** | **SD** | **Count** |
| Basal forebrain | *Gdnf* ^wt/wt^ | young | 563,82 | 256,82 | 10 |
|  |  | old | 404,04 | 146,97 | 8 |
|  | *Gdnf* ^wt/hyper^ | young | 468,15 | 129,04 | 8 |
|  |  | old | 505,78 | 187,27 | 8 |
| Cortex | *Gdnf* ^wt/wt^ | young | 358,20 | 89,00 | 10 |
|  |  | old | 274,35 | 72,12 | 8 |
|  | *Gdnf* ^wt/hyper^ | young | 322,10 | 54,82 | 10 |
|  |  | old | 338,70 | 49,99 | 8 |
| Hippocampus | *Gdnf* ^wt/wt^ | young | 782,71 | 477,00 | 9 |
|  |  | old | 485,38 | 163,31 | 8 |
|  | *Gdnf* ^wt/hyper^ | young | 532,94 | 180,60 | 10 |
|  |  | old | 578,72 | 148,77 | 8 |
| Striatum | *Gdnf* ^wt/wt^ | young | 1005,04 | 298,24 | 10 |
|  |  | old | 853,95 | 340,73 | 8 |
|  | *Gdnf* ^wt/hyper^ | young | 1103,08 | 393,50 | 10 |
|  |  | old | 962,51 | 296,89 | 8 |

SD, standard deviation.

**Supplementary Table 3. Descriptive data of acetylcholine esterase (AChE) activity in the basal forebrain, cortex, hippocampus, and striatum of *Gdnf*^wt/wt^ and *Gdnf*^wt/hyper^ mice.**

| **AChE activity (nmol/min/mg)** | | | | | |
| --- | --- | --- | --- | --- | --- |
| **Brain area** | **Genotype group** | **Age group** | **Mean** | **SD** | **Count** |
| Basal forebrain | *Gdnf* ^wt/wt^ | young | 81,02 | 13,22 | 10 |
|  |  | old | 104,68 | 17,01 | 8 |
|  | *Gdnf* ^wt/hyper^ | young | 87,70 | 8,57 | 8 |
|  |  | old | 105,55 | 15,34 | 8 |
| Cortex | *Gdnf* ^wt/wt^ | young | 16,33 | 1,47 | 10 |
|  |  | old | 15,27 | 1,16 | 8 |
|  | *Gdnf* ^wt/hyper^ | young | 15,12 | 1,85 | 10 |
|  |  | old | 15,78 | 2,15 | 8 |
| Hippocampus | *Gdnf* ^wt/wt^ | young | 29,60 | 11,14 | 9 |
|  |  | old | 24,83 | 4,87 | 8 |
|  | *Gdnf* ^wt/hyper^ | young | 26,74 | 7,09 | 10 |
|  |  | old | 81,02 | 13,22 | 10 |
| Striatum | *Gdnf* ^wt/wt^ | young | 104,68 | 17,01 | 8 |
|  |  | old | 87,70 | 8,57 | 8 |
|  | *Gdnf* ^wt/hyper^ | young | 105,55 | 15,34 | 8 |
|  |  | old | 16,33 | 1,47 | 10 |

SD, standard deviation.

**Supplementary Table 4. Descriptive data of butyrylcholine esterase (BuChE) activity in the basal forebrain, cortex, hippocampus, and striatum of *Gdnf*^wt/wt^ and *Gdnf*^wt/hyper^ mice.**

| **BuChE activity (nmol/min/mg)** | | | | | |
| --- | --- | --- | --- | --- | --- |
| **Brain area** | **Genotype group** | **Age group** | **Mean** | **SD** | **Count** |
| Basal forebrain | *Gdnf* ^wt/wt^ | young | 2,39 | 0,42 | 10 |
|  |  | old | 2,66 | 0,40 | 8 |
|  | *Gdnf* ^wt/hyper^ | young | 2,34 | 0,19 | 8 |
|  |  | old | 2,99 | 0,67 | 8 |
| Cortex | *Gdnf* ^wt/wt^ | young | 2,02 | 0,23 | 10 |
|  |  | old | 1,88 | 0,18 | 8 |
|  | *Gdnf* ^wt/hyper^ | young | 1,93 | 0,12 | 10 |
|  |  | old | 2,04 | 0,26 | 8 |
| Hippocampus | *Gdnf* ^wt/wt^ | young | 2,08 | 0,46 | 9 |
|  |  | old | 1,93 | 0,35 | 8 |
|  | *Gdnf* ^wt/hyper^ | young | 1,94 | 0,37 | 10 |
|  |  | old | 2,41 | 0,50 | 8 |
| Striatum | *Gdnf* ^wt/wt^ | young | 2,95 | 0,23 | 10 |
|  |  | old | 2,97 | 0,41 | 8 |
|  | *Gdnf* ^wt/hyper^ | young | 2,96 | 0,31 | 10 |
|  |  | old | 2,89 | 0,30 | 8 |

SD, standard deviation.

**Supplementary Table 5. Descriptive data of choline level in the basal forebrain, cortex, hippocampus, and striatum of *Gdnf*^wt/wt^ and *Gdnf*^wt/hyper^ mice.**

| **Choline [µmol/g]** | | | | | |
| --- | --- | --- | --- | --- | --- |
| **Brain area** | **Genotype group** | **Age group** | **Mean** | **SD** | **Count** |
| Basal forebrain | *Gdnf* ^wt/wt^ | young | 184,07 | 114,98 | 10 |
|  |  | old | 113,96 | 98,00 | 8 |
|  | *Gdnf* ^wt/hyper^ | young | 123,59 | 70,88 | 8 |
|  |  | old | 160,58 | 125,01 | 8 |
| Cortex | *Gdnf* ^wt/wt^ | young | 83,68 | 28,15 | 10 |
|  |  | old | 65,70 | 29,05 | 8 |
|  | *Gdnf* ^wt/hyper^ | young | 74,42 | 26,50 | 10 |
|  |  | old | 74,55 | 28,94 | 8 |
| Hippocampus | *Gdnf* ^wt/wt^ | young | 204,10 | 147,12 | 9 |
|  |  | old | 93,18 | 95,24 | 8 |
|  | *Gdnf* ^wt/hyper^ | young | 98,98 | 67,72 | 10 |
|  |  | old | 135,93 | 86,95 | 8 |
| Striatum | *Gdnf* ^wt/wt^ | young | 452,96 | 197,76 | 10 |
|  |  | old | 423,28 | 225,35 | 8 |
|  | *Gdnf* ^wt/hyper^ | young | 451,24 | 349,55 | 10 |
|  |  | old | 481,83 | 247,52 | 8 |

SD, standard deviation.

**Supplementary Table 6. Descriptive data of nerve growth factor (NGF) level in the basal forebrain, cortex, hippocampus, and striatum of *Gdnf*^wt/wt^ and *Gdnf*^wt/hyper^ mice.**

| **NGF [ng/mg]** | | | | | |
| --- | --- | --- | --- | --- | --- |
| **Brain area** | **Genotype group** | **Age group** | **Mean** | **SD** | **Count** |
| Basal forebrain | *Gdnf* ^wt/wt^ | young | 0,91 | 0,99 | 10 |
|  |  | old | 1,30 | 0,26 | 8 |
|  | *Gdnf* ^wt/hyper^ | young | 0,90 | 0,12 | 8 |
|  |  | old | 1,88 | 0,96 | 8 |
| Cortex | *Gdnf* ^wt/wt^ | young | 0,61 | 0,16 | 10 |
|  |  | old | 0,68 | 0,13 | 8 |
|  | *Gdnf* ^wt/hyper^ | young | 0,48 | 0,13 | 10 |
|  |  | old | 0,69 | 0,08 | 8 |
| Hippocampus | *Gdnf* ^wt/wt^ | young | 0,64 | 0,17 | 9 |
|  |  | old | 0,64 | 0,21 | 8 |
|  | *Gdnf* ^wt/hyper^ | young | 0,59 | 0,14 | 10 |
|  |  | old | 0,73 | 0,23 | 8 |
| Striatum | *Gdnf* ^wt/wt^ | young | 1,26 | 0,23 | 10 |
|  |  | old | 2,37 | 0,75 | 8 |
|  | *Gdnf* ^wt/hyper^ | young | 1,33 | 0,35 | 10 |
|  |  | old | 2,61 | 1,19 | 8 |

SD, standard deviation.


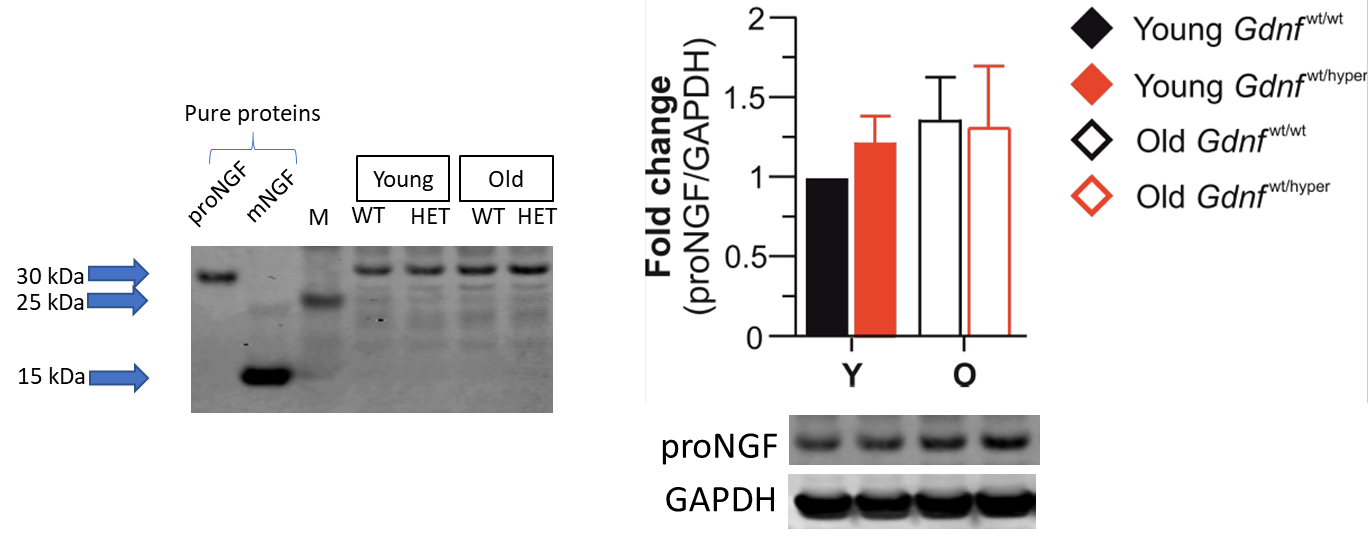


**Supplementary Figure 1. NGF levels in cortical tissues of *Gdnf*^wt/wt^ and *Gdnf*^wt/hyper^ mice**. Western blot analysis showed that proNGF is the major form of NGF observed in the cortex. Brain tissue homogenates were prepared in RIPA buffer (MerckMillipore, #20-188), resolved in 4-12% gels (Thermofisher, #NP0322BOX) and transferred on to PVDF membrane (Thermofisher, #22860). Blots were blocked (Thermofisher, #37587) and probed with rabbit anti-NGF antibody (Alomone labs, #AN-240) and further detected with IRDye® 800CW Donkey anti-Rabbit IgG Secondary Antibody (LI-COR biosciences, #926-32213) and visualized in Odyssey® CLx Imaging System (LI-COR biosciences). Densitometric data are shown as ratio between proNGF/GAPDH and presented as mean fold change ± SEM. N = 4 for each group of samples. Abbreviations: M = molecular weight marker; O = old; Y = young.
